# New mechanisms involving the EGFR and FGF15/19 systems in liver regeneration and carcinogenesis

**DOI:** 10.1186/2047-783X-19-S1-S16

**Published:** 2014-06-19

**Authors:** Carmen Berasain, Matías A Avila

**Affiliations:** 1 Division of Hepatology and Gene Therapy, CIMA. University of Navarra, Pamplona, 31008, Spain; 2 CIBERehd, University of Navarra Clinic, Pamplona, 31008, Spain

## 

**Figure 1:**
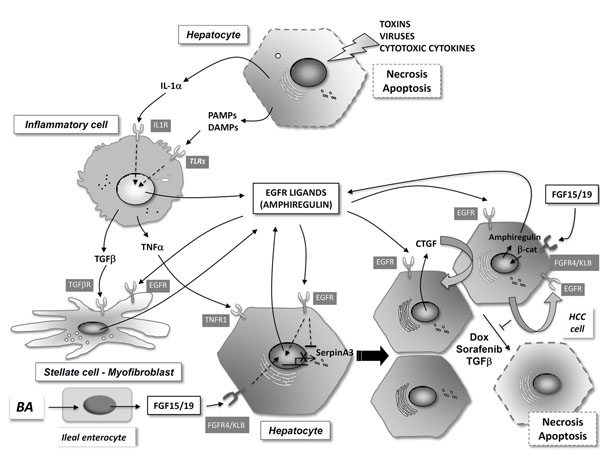


In response to tissue injury the liver mounts a robust protective and regenerative response aimed at the restoration of the lost or damaged parenchyma. Different molecules have been identified to play important roles in the coordination of the reparative process after liver damage. These can be of varied molecular nature, including inflammatory mediators like cytokines, growth factors such as epidermal growth factor receptor (EGFR) ligands, and metabolites like bile acids (BAs) [1]. These molecules establish complex crosstalks involving different liver cell types, and in some instances also extrahepatic tissues, ultimately leading to the survival and proliferation of hepatocytes and non-parenchymal cells. The identification of biologically relevant and potent effectors of liver tissue repair may provide new therapeutic tools to treat acute liver failure, allow more extensive hepatic resection in oncologic surgery, and utilize smaller or marginal liver grafts. BAs are emerging as key metabolites involved in liver regeneration after partial hepatectomy (PH). Their intrahepatic and serum levels transiently increase after liver tissue resection, and the preservation of the enterohepatic circulation of BAs seems to be essential for liver regeneration [2,3]. BAs can interact with the cell surface receptor TGR5 and also with the nuclear receptor FXR, and both interactions are important for liver regeneration to proceed normally. BAs are taken up from the gut lumen in the distal ileum, and return to the liver through the portal circulation. It has been known for many years that portal blood containes essential components for liver regeneration after PH, and BAs could be one of these components that promote hepatic growth [1]. Importantly, during their passage through the ileal enterocytes BAs also bind and activate their FXR receptor. One of the FXR target genes in the ileal enterocytes is fibroblast growth factor 19 (FGF19, FGF15 in rodents). FGF15/19 has been recently characterized as an important hormone with effects similar to those of insulin, and a potent inhibitory action on liver BA synthesis. We have identified a new role for FGF15/19 in liver regeneration after PH. FGF15/19 deficient mice showed reduced survival after liver tissue resection, attributable to their inability to downregulate BA synthesis after PH and the consequent toxic cholestasis [3]. Importantly, we could also demonstrate that FGF15/19 was also essential for the growth-promoting effects of BAs on liver parenchymal cells and cholangiocytes. Indeed, the proliferative effects of a cholate-supplemented diet observed in wild type mice were markedly attenuated in FGF15/19 deficient animals [3]**(**Figure 1**).** These findings attest to the importance of FGF15/19 in liver regeneration, and led us to test the efficacy of exogenously administered FGF15 in mice subjected to extensive liver resection (85% PH), a clinically relevant model of the small-for-size syndrome. Overexpression of FGF15 in the liver of wild type mice, by means of an adenoviral vector encompassing FGF15 cDNA, significantly enhanced mouse survival. While all mice infected with a control adenovirus died 24 h after 85% PH, almost 50% of mice infected with the FGF15 expressing adenoviral vector survived the intervention. These findings support the potential application of FGF15/19 as a liver pro-regenerative strategy in the acute setting.

There are two important aspects regarding the signals and pathways activated during liver injury and regeneration. One is their extensive crosstalk, with frequent mutual influences on the expression and activities of the different growth factors and cytokines. The other is the common involvement of many of these mediators and pathways in the liver carcinogenic process, when the reparative reaction goes awry in the setting of chronic liver injury. This has been cogently demonstrated for the epidermal growth factor receptor (EGFR) signaling system. Normal hepatocytes express high levels of EGFR, EGFR activation contributes significantly to hepatocellular survival and proliferation during injury and regeneration, and also plays a fundamental role in the negative regulation of liver acute phase response triggered by inflammatory cytokines [4]. Amphiregulin (AR) is one of the ligands of the EGFR which expression is markedly up-regulated during liver inflammation and injury (Figure 1). AR plays a non-redundant role among EGFR ligands in hepatocyte proliferation and survival in different models of liver injury and regeneration, including Fas activation, CCl_4_ administration, ischemia-reperfusion and PH [5]. Interestingly, AR is also essential for the attenuation of the liver acute phase response, contributing to the repression of acute phase proteins such as α1-antichymotrypsin, also known as serpinA3 [4,5]. Persistent AR up-regulation has been detected in chronic liver injury, and this growth factor plays an important role in hepatic fibrogenesis, a consequence of the dysregulated wound healing response occuring during chronic liver damage [5]. Furthermore, AR mediates an autocrine stimulatory loop contributing to the neoplastic properties of hepatocellular carcinoma (HCC) cells through the stimulation of the EGFR [4]. Importantly, AR contributes to the resistance of HCC cells to the growth inhibitory and pro-apoptotic actions of transforming growth factor β (TGFβ), conventional cytotoxic drugs like doxorubicin (Dox), and most importantly targeted anti-HCC agents like sorafenib (Figure 1). Regarding the mechanisms leading to AR up-regulation in HCC cells, we found that AR gene expression could be induced by FGF19, which in turn is frequently overexpressed in human HCC cells [5]. This stimulatory effect of FGF19 on AR gene transcription was mediated thorugh the stimulation of β-catenin (b-cat) signaling. This is an example of the extensive crosstalk among different growth factors in liver injury and carcinogenesis alluded above.

Finally, we could also identify a novel pro-tumorigenic mechanism activated by the AR/EGFR signaling system in liver cancer development. As previously mentioned, AR plays a fundamental role in the negative control of the acute phase reaction, and in the down-regulation of acute phase proteins during liver regeneration, counteracting the effects of inflammatory cytokines like oncostatin M (OSM). One of the most dysregulated acute phase genes in AR deficient mice undergoing an acute phase was serpinA3, a serin-protease inhibitor secreted by hepatocytes during inflammation [6]. We found that serpinA3 expression was markedly reduced in HCC cells due to an AR/EGFR autocrine loop (Figure 1), and importantly reduced serpinA3 expression was consistently found in human HCC tissues [6]. Intriguingly, serpinA3 was also detected in the nucleus of hepatocytes, and restitution of the expression of this gene significantly blunted HCC cell proliferation and the growth of HCC xenografts in nude mice [6]. Interestingly, from a mechanistically point of view, we could demonstrate that nuclear serpinA3 forms polymeric aggregates that tightly bind chromatin, inducing a condensed status that is not compatible with DNA synthesis and cell proliferation. These findings provide a mechanistic explanation to the early observations of reduced acute phase gene expression during liver regeneration, and identify serpinA3 as an important regulator of hepatocellular proliferation, which is lost during liver cancer development. Together, our observations provide novel insights into the mechanisms of liver repair and regeneration. We also identify new pro-regenerative agents with potential therapeutic application in an acute setting, as well as novel targets for antitumoral intervention.
